# Microsatellite instability and mismatch repair deficiency prevalence among Hispanic/Latino individuals with colorectal cancer: a systematic review and meta-analysis

**DOI:** 10.1007/s00384-026-05146-2

**Published:** 2026-05-21

**Authors:** Gabriela Guerron-Gomez, Daniel F. Mendivelso-González, Viviana Chaves-Cabezas, Juan José Chaves, Julián C. Riaño-Moreno, Rafael Parra-Medina

**Affiliations:** 1 Research Institute, Fundación Universitaria de Ciencias de la Salud (FUCS), Cra. 54 #67a80, Bogotá, Colombia; 2 Department of Pathology, Instituto Nacional de Cancerología, Bogotá, Colombia; 3 Department of Pathology, Fundación Universitaria de Ciencias de La Salud (FUCS), Bogotá, Colombia; 4 Department of Medicine, Norwalk Hospital, Yale School of Medicine, Norwalk, CT USA; 5 Faculty of Medicine, Cooperative University of Colombia, Villavicencio, Colombia

**Keywords:** MSI-high, Colorectal carcinoma, Mismatch repair, Hispanic/Latino population

## Abstract

**Background:**

Colorectal cancer (CRC) is the third most common cancer globally, with rising cases in Latin America. MSI-H and MMRd play key roles in CRC, but data on their prevalence in Hispanic/Latino populations are limited. This study evaluates these biomarkers in the region.

**Methods:**

A systematic review and meta-analysis were conducted following PRISMA guidelinesin Medline, Virtual Health Library, Scopus, and Web of Science. Random-effects models were used to estimate pooled prevalence due to expected heterogeneity between studies. Studies (cohort and cross-sectional) that evaluated MMRd and/or MSI-H through IHC and PCR techniques in Hispanic/Latino individuals with colorectal cancer (whether sporadic, associated with Lynch Syndrome, or other forms), residing in Latin American countries or elsewhere, were included.

**Results:**

A total of 52 studies including 10,596 patients were included. The pooled prevalence of mismatch repair deficiency (MMRd) and microsatellite instability-high (MSI-H) in Hispanic/Latino populations was 15% (95% CI: 10%–20%; I^2^ = 89.6%) and 18% (95% CI: 13%–24%; I^2^ = 84.0%), respectively. Costa Rica and Mexico had the highest MMRd prevalence (30% and 24%), while Uruguay showed the highest MSI-H prevalence (45%). MSI-H was significantly associated with female sex (OR: 1.83) and right-sided tumors (OR: 8.16). MMRd was associated with right-sided tumors compared with the rectum (OR: 1.73) and the left colon (OR: 5.65).

**Conclusions:**

This meta-analysis underscores the unique prevalence of MMRd and MSI-H in Hispanics, highlighting regional variations and the need for broader representation.

**Supplementary Information:**

The online version contains supplementary material available at 10.1007/s00384-026-05146-2.

## Introduction

According to GLOBOCAN 2022, colorectal cancer (CRC) is the third most common cancer globally, with 1,926,425 new cases reported and an age-standardized rate of 18.4 per 100,000 people. It remains the second leading cause of cancer-related deaths, accounting for 903,859 fatalities in 2022. Notably, CRC incidence and mortality rates show significant geographical variation [1].

In Latin American countries, CRC mortality increased by 20.5% between 1990 and 2019, contrasting with declining rates in high-income countries [2]. Also, CRC incidence correlates with the Human Development Index (HDI), higher HDI levels are linked to greater incidence [3]. Although some Hispanic/Latino countries have achieved high HDI status due to economic progress, the region remains marked by income inequality and social vulnerability [2]. These disparities contribute to uneven access to biomarker technologies and treatments, creating significant heterogeneity in CRC diagnosis and management across the region [3].

Different biomarkers have been established for CRC, of which microsatellite instability (MSI) is one of the most recognized and clinically validated. MSI is crucial for screening hereditary syndromes, predicting prognosis, and guiding treatment decisions [4]. CRC can be classified based on MSI status into high microsatellite instability (MSI-H), low microsatellite instability (MSI-L), or microsatellite stable (MSS) [5]. MSI-H is a hypermutable phenotype caused by a defective DNA mismatch repair (MMR) system, often resulting from the inactivation of the MMR genes *MSH2*, *MLH1*, *MSH6*, and *PMS2*, which prevents the correction of insertion or deletion errors during DNA replication [6].

MSI-H CRC exhibits distinct histopathological features, including tumor-infiltrating lymphocytes (TILs), absence of dirty necrosis, Crohn-like reactions, right-sided tumor location, mucinous differentiation (focal or extensive), and either well- or poorly differentiated morphology. Additionally, MSI-H tumors are associated with a high neoantigen burden [7–9]. These characteristics render MSI-H CRC tumors highly responsive to immune checkpoint inhibitors, making them excellent candidates for immunotherapy [4, 10].

Approximately 15–17% of all CRC cases exhibit MSI-H. MSI can result from somatic alterations in MMR genes, such as pathogenic or likely pathogenic (P/LP) variants or MLH1 promoter methylation, which are commonly associated with sporadic CRC. Most of these cases (75%–80%) are linked to acquired *MLH1* promoter methylation and the CpG island methylator phenotype (CIMP). Alternatively, MSI can arise from germline P/LP variants in MMR genes or EPCAM deletions, accounting for 2%–3% of CRC cases and characterizing Lynch syndrome [6].

Given the critical role of MSI in clinical decision-making for CRC, particularly in guiding chemotherapy and immunotherapy strategies, the American Society of Clinical Oncology (ASCO) underscores the importance of MSI testing. As a result, MSI status evaluation is now recommended for all CRC cases [10, 11].

MSI-H and MMR deficiency (MMRd) are closely related biomarkers that reflect defects in the DNA mismatch repair system in CRC. MSI can be assessed indirectly through MMR protein expression using immunohistochemistry (IHC), which screens for the loss of MMR proteins MLH1, MSH2, MSH6, and PMS2. MMRd is defined as the complete loss of at least one protein, while MMR proficiency (MMRp) is indicated by positive staining for all four proteins [12, 13]. Alternatively, MSI can be directly detected using polymerase chain reaction (PCR-MSI), which evaluates microsatellite markers such as BAT25, BAT26, D2S123, D5S346, and D17S2720. PCR is considered positive when instability is identified in at least two markers [14–17]. Although the MSI-H and MMRd are highly concordant, discordant results have been reported in a minority of cases due to technical and biological factors [4, 11, 12, 15, 18].

IHC is widely used for MSI detection due to its lower cost, faster turnaround time, and high accuracy, achieving a concordance rate of 98.4% between MSI-H and MMRd expression in CRC. [18] While PCR-MSI is part of the biomarker routine in some centers, it is primarily recommended for cases with discordant IHC results, as it provides a more detailed MSI assessment, particularly when discrepancies could influence treatment decisions [15, 19, 20]. Recently, Next Generation Sequencing (NGS-MSI) has gained attention with the increasing adoption of Comprehensive Genomic Profiling (CGP) in cancer biomarker analysis [12]. However, the 2022 CAP guidelines prioritize PCR and IHC-MMR over NGS-MSI for colorectal carcinoma, citing limited evidence for NGS, its high cost, and reduced accessibility in developing countries [21].

Some studies have reported varying biomarker prevalences across cancer types and patient populations [22–24]. Latin America and the Caribbean exhibit unique genetic backgrounds, cultural behaviors, environmental exposures, and socioeconomic heterogeneity, all of which may influence the prevalence of MSI alterations in CRC [25]. While studies suggest that the prevalence of MSI in CRC among Hispanic/Latino individuals is comparable to other ethnic groups [26, 27] comprehensive regional data remain scarce. This systematic review and meta-analysis aim to summarize the clinical characteristics and estimate the prevalence of MMRd and MSI-H biomarkers (MMRd/MSI-H) in CRC populations from Latin American countries and Hispanic individuals living outside the region.

## Materials and methods

This systematic review was conducted following the PRISMA (Preferred Reporting Items for Systematic Reviews and Meta-Analyses) guidelines [28]. The protocol was registered in PROSPERO, the International Prospective Register of Systematic Reviews, under the identifier CRD42024573607.

### Information sources and search strategy

A comprehensive search strategy was conducted across Medline, Virtual Health Library, Scopus, and Web of Science. The term Hispanic/Latino was defined to include individuals of Spanish ancestry in the United States and all Latin American countries, including Brazil. As a result, the search incorporated the term "Hispanic or Latino." Specific Medical Subject Headings (MeSH) terms such as "Colonic Neoplasms," "DNA Mismatch Repair," and "Microsatellite Instability" were also used (Supplementary Table 1). Grey literature was accessed through Google Scholar, and the final database search was completed on May 31, 2024. The language was restricted to English, Spanish and Portuguese. Additionally, a manual review of reference lists from selected articles was conducted.

### Inclusion criteria

The inclusion criteria for this systematic review encompassed original cohort and cross-sectional studies that evaluated MMRd or MSI-H through IHC and PCR techniques in Hispanic/Latino individuals with colorectal cancer (whether sporadic, associated with Lynch Syndrome, or other forms), residing in Latin American countries or elsewhere. NGS-MSI was not included due to the pre-defined limitations in accessing NGS technologies in Hispanic/Latino populations. The studies required to have performed IHC and/or PCR-MSI testing on tumor tissue. There were no language restrictions, and the search was conducted up to May 2024.

For the purposes of this review, Hispanic/Latino populations were defined according to the classifications used in the original studies. This included individuals from Latin American countries as well as populations identified as Hispanic or Latino in studies conducted in other regions, particularly the United States (US). In most studies, ethnicity was determined by geographic origin or self-reported ethnicity. Given the complex genetic ancestry and admixture characteristic of Latin American populations, which includes varying contributions of Indigenous American, European, and African ancestries, the term Hispanic/Latino was used as reported by the original authors rather than representing a genetically homogeneous group [22, 25, 29].

### Exclusion criteria

The exclusion criteria for this review were as follows: a) Studies that assessed fewer than 4 MMR proteins (MLH1, MSH2, MSH6, PMS2), b) Studies that presented inconsistencies between the text and the data reported in tables, c) Studies that did not differentiate molecular alteration information between Hispanic/Latino individuals and other ethnic groups, d) Studies that evaluated MMRd/MSI-H status exclusively in a subset of individuals with a specific molecular alteration.

### Study selection

Articles selected were assessed by four primary reviewers (GGG, DMG, JCC, VCC) that independently screened the titles and abstracts of the studies to determine eligibility. The same reviewers then conducted a thorough assessment of the full texts of the selected articles, excluding those that did not meet the established criteria. References of identified studies were reviewed to find additional relevant articles. Discrepancies among the reviewers were resolved by a fifth author (RPM or JCR).

### Data collection process and data extraction

The following information was extracted from each article, when available: authors, year of publication, country, study period, population size, age, sex, MMR/MSI assessment methods, prevalence of MMRd/MSI-H, affected MMR proteins, and tumor size, location, histological type, and stage.

### Risk of bias and applicability

Risk of bias was assessed using the Joanna Briggs Institute (JBI) Critical Appraisal Checklist for cohort and cross-sectional studies was implemented [30]. Three authors (GGG, DFMG, RPM) answered eight questions for cross-sectional studies and eleven questions for cohort studies. Each question was rated as ‘yes’, no’, ‘unclear’, or ‘not applicable’. A scoring system was applied by calculating the proportion of “Yes” responses. Studies with ≥ 70% “Yes” responses were considered at low risk of bias, those with 50–69% at moderate risk, and those with < 50% at high risk.

### Summary measures

The primary outcomes of this study were the prevalence of MMRd/MSI-H tumors among cases of colorectal cancer in Hispanic/Latino individuals. Prevalence was calculated as the proportion of MMRd/MSI-H cases relative to the total number of tests conducted, with country-specific prevalence also determined. Furthermore, ORs for MMRd/MSI-H were calculated in relation to age, gender, tumor stage, location, and histological subtype.

### Data synthesis and analysis

Quantitative analyses of the included studies were conducted in R version 4.3.1 using the meta and metafor packages. Two separate meta-analyses with a random-effects model were performed to estimate the prevalence of MMRd/MSI-H among Hispanic/Latino individuals with colorectal cancer. Additionally, we employed ORs with corresponding confidence intervals to assess the correlation between MMRd/MSI-H and various clinicopathological features. Meta-analyses were conducted using Review Manager 5.4.1 software from the Cochrane Collaboration. A random-effects model was selected expecting substantial clinical and methodological heterogeneity across studies. Heterogeneity was assessed with Cochran's Q and quantified with the I^2^ statistic, interpreted as low (≤ 25%), moderate (25–75%), or high (> 75%). A significance level of 5% was used, and heterogeneity was assessed according to sample size, country, and study design. Geographic heterogeneity was explored descriptively through country-based subgroups displayed in the forest plots. Formal subgroup or meta-regression analyses according to diagnostic modality, tumor stage, or Lynch syndrome inclusion were not performed because the required study-level data were incomplete, inconsistently reported, or not directly comparable across reports.

MMRd and MSI-H were conceptually grouped as complementary markers of mismatch repair dysfunction in colorectal cancer, because both are used to identify tumors with defective mismatch repair biology and have relevance for molecular classification, Lynch syndrome screening, prognosis, and therapeutic decision-making [31, 32]. However, they were analyzed separately because they are not identical biomarkers, and clinically meaningful discordance has been documented despite their high overall concordance [15]. Discordant cases could not be reclassified at the review level because the primary studies did not consistently report paired assay-level results or explicit adjudication algorithms; therefore, cases were synthesized according to the endpoint reported by the original authors [15, 32].

### Sensitivity analysis

To assess the robustness of the pooled prevalence estimates in the presence of substantial heterogeneity, we performed leave-one-out sensitivity analyses for both the MMRd and MSI-H meta-analyses using the metafor package in R. Each study was sequentially omitted, and the random-effects model was re-estimated using restricted maximum likelihood (REML) under the same analytical assumptions as in the primary analyses. The leave-one-out analyses were presented as Figure S1 (MMRd) and Figure S2 (MSI-H).

## Results

### Results of the search and screening

**Fig. 1:**
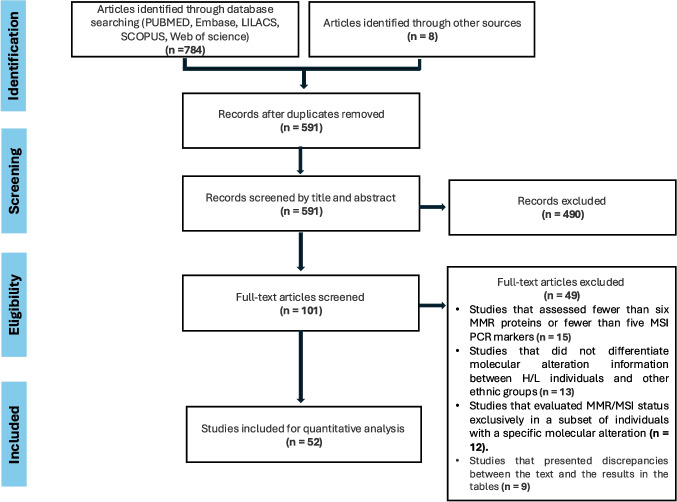
Search strategy

The PRISMA flow diagram, shown in Fig. 1 summarizes the search process. After removing duplicates, 591 records were screened based on their titles and abstracts. Following this initial screening, 101 articles were selected for full-text assessment, and 49 studies were excluded because they did not meet the inclusion criteria. Finally, 52 studies were included in the review, 40 cross-sectional studies and 12 cohort studies. Of these, 22 studies evaluated MMR only, 24 focused on MSI only, and 4 assessed both MMR and MSI. Most of the studies were from Brazil, with 18 articles, followed by 7 studies on Hispanic individuals living in the United States. The countries of the other participants were Chile (*n* = 5), Colombia (*n* = 5), Puerto Rico (*n* = 5), Argentina (*n* = 3), Mexico (*n* = 3), Peru (*n* = 4), Dominican Republic (*n* = 1), Costa Rica (*n* = 1), Ecuador (*n* = 1), and Paraguay (*n* = 1).

### General clinical information

Clinical data were extracted from 52 articles, encompassing a total of 10,596 individuals. Among studies evaluating MMRd, the mean age of participants was 58.71 years. Of these, 63.4% (1928/3041) were male, 69.7% (1667/2391) had tumors located in the left colon or rectum, 52.2% (70/134) presented with non-mucinous adenocarcinoma histology, and 55.8% (663/1188) had stage III-IV tumors (Table 1). In studies assessing MSI status, the mean age was 64.17 years. Of the participants, 71.8% (1039/1448) were male, 67.4% (807/1198) had tumors in the left colon or rectum, 72.6% (98/135) displayed a non-mucinous adenocarcinoma histological subtype, and 53.9% (96/178) had stage III-IV tumors (Table 2).

**Table 1:** Clinical characteristics of patients included with MMR status by country

MMR Status														
Author/Year	Country/Region	Type of study	Type of test performed	Mean age	Total		Gender				Location			
					dMMR	pMMR	Male		Female		Right colon		Left colon/Rectum	
							dMMR	pMMR	dMMR	pMMR	dMMR	pMMR	dMMR	pMMR
Antelo et al. 2019 [33]	Argentina	Cross-sectional	PCR & IHC	34.52	21	81	10	41	11	40	12	14	9	67
Schmitz et al. 2014 [34]	Argentina	Cross-sectional	IHC	57	16	41	10	56	6	43	-	-	-	-
Azambuja et al. 2023 [35]	Brazil	Cohort	PCR & IHC	64	8	55	-	-	-	-	-	-	-	-
Berardinelli et al. 2018 [36]	Brazil	Cohort	PCR & IHC	57.8	102	886	528	-	-	-	238	-	520	-
De Freitas et al. 2015 [37]	Brazil	Cross-sectional	PCR & IHC	68	8	53	8	-	4	-	-	6	5	-
Germini et al. 2019 [38]	Brazil	Cohort	PCR & IHC	65.4	4	57	-	-	-	-	-	-	-	-
Gomes et al. 2021 [39]	Brazil	Cross-sectional	IHC	56.8	78	920	-	-	-	-	-	-	-	-
Alex et al. 2017 [40]	Brazil	Cross-sectional	IHC	52	41	84	28	38	13	46	26	21	15	63
Paula Simedan Vila et al. 2023 [41]	Brazil	Cross-sectional	IHC	60	43	327	20	179	23	148	-	-	-	-
Sunagua Aruquipa et al. 2024 [42]	Brazil	Cross-sectional	PCR & IHC	63,7	14	844	-	-	-	-	-	-	-	-
Gómez-Rodríguez et al. 2021 [43]	Colombia	Cross-sectional	IHC	69	12	74	9	36	3	38	8	37	2	37
Shamek et al. 2016 [44]	Colombia & USA	Cross-sectional	IHC	63	11	52	6	18	5	16	-	-	-	-
Allan et al. 2020 [45]	Costa Rica	Cohort	IHC	66.5	165	388	120	268	47	120	72	42	92	346
Quezada-Diaz et al. 2022 [46]	Chile	Cross-sectional	IHC	-	8	57	-	-	-	-	5	16	3	41
Bacilio et al. 2018 [47]	Ecuador	Cross-sectional	IHC	66.2	182	58	71	20	111	38	-	-	-	-
Lopez-Correa et al. 2018 [48]	Mexico	Cross-sectional	IHC	59	43	159	24	95	19	64	17	18	22	140
De De Mexico et al. 2022 [49]	Mexico	Cross-sectional	IHC	65	39	105	21	57	18	48	27	35	12	70
Fleitas-Kanonnikoff et al. 2019 [50]	Paraguay	Cohort	IHC	52	5	31	2	21	3	10	2	12	3	19
Egoavil et al. 2011 [51]	Peru	Cross-sectional	IHC	59.3	35	54	-	-	-	-	-	-	-	-
Carbajal et al. 2014 [52]	Peru	Cross-sectional	IHC	-	1	2	-	-	-	-	-	-	-	-
De Jesus-Monge et al. 2010 [53]	Puerto Rico	Cross-sectional	PCR & IHC	59.9	7	157	3	67	4	90	-	-	-	-
Sierra et al. 2021 [54]	Puerto Rico	Cross-sectional	IHC & PCR (Only MMR reported)	65,8	3	26	11	161	24	121	25	91	10	191
Cruz-Correa et al. 2015 [55]	Puerto rico & Dominican Republic	Cross-sectional	PCR & IHC	-	13	45	-	-	-	-	-	-	-	-
Barrows et al. 2017 [56]	U.S.A hispanics	Cross-sectional	IHC	53,9	14	88	-	-	-	-	-	-	-	-
Berera et al. 2016 [57]	U.S.A hispanics	Cross-sectional	IHC	60	13	90	-	-	-	-	-	-	-	-
Ricker et al. 2017 [58]	U.S.A hispanics	Cross-sectional	PCR & IHC	53,7	21	140	-	-	-	-	-	-	-	-
Fangman et al. 2021 [59]	U.S.A hispanics	Cohort	IHC	42	28	106	-	-	-	-	-	-	-	-
Gupta et al. 2010 [27]	U.S.A hispanics	Cohort	PCR & IHC	57	14	85	-	-	-	-	-	-	-	-
Hoffman et al. 2018 [60]	U.S.A hispanics	Cross-sectional	IHC	61.3	4	35	-	-	-	-	-	-	-	-
Reverón et al. 2018 [61]	U.S.A hispanics	Cross-sectional	IHC	-	6	89	-	-	-	-	-	-	-	-

MMR Status												
Author/Year	Histology subtype				Stage							
	Mucinous adenocarcinoma		Conventional adenocarcinoma		I		II		III		IV	
	dMMR	pMMR	dMMR	pMMR	dMMR	pMMR	dMMR	pMMR	dMMR	pMMR	dMMR	pMMR
Antelo et al. 2019 [33]	-	-	-	-	I—II: MMRd = 11, MMRp = 26, III—IV: MMRd = 10, MMRp = 55							
Schmitz et al. 2014 [34]	-	-	-	-	-	-	-	-	-	-	-	-
Azambuja et al. 2023 [35]	-	-	-	-	-	-	-	-	-	-	-	-
Berardinelli et al. 2018 [36]	-	-	-	-	-	-	-	-	-	-	-	-
De Freitas et al. 2015 [37]	1	-	-	-	3	-	4	-	-	-	2	-
Germini et al. 2019 [38]	-	-	-	-	-	-	-	-	-	-	-	-
Gomes et al. 2021 [39]	-	-	-	-	-	-	-	-	-	-	-	-
Alex et al. 2017 [40]	17	13	-	-	-	-	-	-	-	-	-	-
Paula Simedan Vila et al. 2023 [41]	-	-	-	-	-	-	-	-	-	-	-	-
Sunagua Aruquipa et al. 2024 [42]	-	-	-	-	-	-	-	-	-	-	-	-
Gómez-Rodríguez et al. 2021 [43]	3	10	8	62	-	-	-	-	-	-	-	-
Shamekh et al. 2016 [44]	-	-	-	-	-	-	-	-	-	-	-	-
Allan et al. 2020 [45]	-	-	-	-	20	12	36	163	58	141	52	72
Quezada-Diaz et al. 2022 [46]	-	-	-	-	-	2	2	20	5	23	1	12
Bacilio et al. 2018 [47]	-	-	-	-	27	9	54	22	45	14	56	13
Lopez-Correa et al. 2018 [48]	6	14	-	-	5	8	11	15	9	21	4	10
De De Mexico et al. 2022 [49]	-	-	-	-	-	-	-	-	-	-	-	-
Fleitas-Kanonnikoff et al. 2019 [50]	-	-	-	-	-	-	-	-	-	-	-	-
Egoavil et al. 2011 [51]	-	-	-	-	-	-	-	-	-	-	-	-
Carbajal et al. 2014 [52]	-	-	-	-	-	-	-	-	-	-	-	-
De Jesus-Monge et al. 2010 [53]	-	-	-	-	-	9	3	63	3	43	1	13
Sierra et al. 2021 [54]	-	-	-	-	-	-	-	-	-	-	-	-
Cruz-Correa et al. 2015 [55]	-	-	-	-	-	-	-	-	-	-	-	-
Barrows et al. 2017 [56]	-	-	-	-	-	-	-	-	-	-	-	-
Berera et al. 2016 [57]	-	-	-	-	-	-	-	-	-	-	-	-
Ricker et al. 2017 [58]	-	-	-	-	-	-	-	-	-	-	-	-
Fangman et al. 2021 [59]	-	-	-	-	-	-	-	-	-	-	-	-
Gupta et al. 2010 [27]	-	-	-	-	-	-	-	-	-	-	-	-
Hoffman et al. 2018 [60]	-	-	-	-	-	-	-	-	-	-	-	-
Reverón et al. 2018 [61]	-	-	-	-	-	-	-	-	-	-	-	-

**Table 2:** Clinical characteristics of patients included with MSI status by country

MSI Status														
Author/Year	Country/Region	Type of study	Type of test performed	Mean age	Total		Sex				Location			
					MSI	MSS	Male		Female		Right colon		Left colon	
							MSI	MSS	MSI	MSS	MSI	MSS	MSI	MSS
Lerda et al. 2019 [62]	Argentina	Cross-sectional	PCR	60.6	4	27	3	16	1	11	-	-	-	-
Sánchez et al. 2020 [63]	Argentina	Cross-sectional	PCR & IHC	-	11	99	-	-	-	-	-	-	-	-
Anacleto et al. 2005 [64]	Brazil	Cross-sectional	PCR	63	14	92	-	-	-	-	13	9	5	65
Berardinelli et al. 2018 [36]	Brazil	Cohort	PCR & IHC	57.8	106	907	528	-	-	-	-	238	520	-
da Silva et al. 2015 [65]	Brazil	Cross-sectional	PCR	-	43	123	-	-	-	-	-	-	-	-
De Freitas et al. 2015 [37]	Brazil	Cross-sectional	PCR & IHC	68	12	49	8	-	4	-	6	-	5	-
de Oliveira et al. 2023 [66]	Brazil	Cross-sectional	PCR & IHC	-	10	112	7	57	55	3	40	8	2	72
dos Santos et al. 2019 [67]	Brazil	Cohort	PCR	61	12	78	-	-	-	-	-	-	-	-
Moraes Losso et al. 2012 [68]	Brazil	Cross-sectional	PCR	-	16	22	-	-	-	-	-	-	-	-
Leite et al. 2010 [69]	Brazil	Cross-sectional	PCR	-	15	51	-	-	-	-	-	-	-	-
Proença et al. 2018 [70]	Brazil	Cross-sectional	PCR	63	7	36	-	-	-	-	-	-	-	-
Rasuck et al. 2012 [71]	Brazil	Cross-sectional	PCR & MLPA	-	16	61	-	-	-	-	-	-	-	-
Santos et al. 2024 [72]	Brazil	Cohort	PCR & Sanger	-	7	200	5	110	2	94	-	4	3	-
Afanador et al. 2022 [73]	Colombia	Cross-sectional	PCR	-	12	32	-	-	-	-	-	-	-	-
Cardenas et al. 2008 [74]	Colombia	Cross-sectional	PCR	-	3	8	5	-	6	-	-	-	-	-
Montenegro et al. 2006 [75]	Colombia	Cross-sectional	PCR	-	11	30	-	-	-	-	-	-	-	-
Alvarez et al. 2021 [76]	Chile	Cohort	PCR	-	74	342	5	110	2	94	4	-	3	-
Hurtado et al. 2015 [77]	Chile	Cross-sectional	PCR	-	15	43	7	21	8	22	11	-	4	-
MAríA Wielandt et al. 2017 [78]	Chile	Cross sectional	PCR & IHC	70	9	44	4	25	5	19	12	8	1	32
Wielandt et al. 2020 [79]	Chile	Cohort	PCR	66	15	88	8	59	7	29	20	13	2	68
Jordi et al. n.d. [80]	Mexico	Cross-sectional	PCR	-	10	20	-	-	-	-	5	0	5	20
Ortiz et al. 2016 [81]	Peru	Cross-sectional	Electrophoresis and PCR	-	11	17	-	-	-	-	-	-	-	-
Perez-Mayoral et al. 2023 [82]	Puerto Rico	Cross-sectional	PCR & IHC	-	6	186	-	-	-	-	-	-	-	-
Sierra et al. 2021 [54]	Puerto Rico	Cross-sectional	PCR & IHC	-	3	26	-	-	-	-	-	-	-	-
Cruz-Correa et al. 2015 [55]	Puerto rico & Dominican Republic	Cross-sectional	PCR & IHC	-	8	22	-	-	-	-	-	-	-	-
Vital et al. 2023 [83]	Uruguay	Cross-sectional	PCR	61.7	54	54	31	30	23	24	-	-	-	-
Antelo et al. 2019 [33]	U.S.A hispanics	Cross-sectional	PCR & IHC	-	54	48	-	-	-	-	-	-	-	-
Gupta et al. 2010 [27]	U.S.A hispanics	Cohort	PCR & IHC	-	11	100	-	-	-	-	-	-	-	-

MSI Status												
Author/Year	Histology subtype				Stage							
	Mucinous adenocarcinoma		Conventional adenocarcinoma		I		II		III		IV	
	MSI	MSS	MSI	MSS	MSI	MSS	MSI	MSS	MSI	MSS	MSI	MSS
Lerda et al. 2019 [62]	-	-	-	-	-	-	-	-	-	-	-	-
Sánchez et al. 2020 [63]	-	-	-	-	-	-	-	-	-	-	-	-
Anacleto et al. 2005 [64]	-	-	-	-	-	-	-	-	-	-	-	-
Berardinelli et al. 2018 [36]	-	-	-	-	-	-	-	-	-	-	-	-
da Silva et al. 2015 [65]	-	-	-	-	-	-	-	-	-	-	-	-
De Freitas et al. 2015 [37]	1	-	-	-	3		4	-	-	-	2	-
de Oliveira et al. 2023 [66]	3	18	6	92	-	-	-	-	-	-	-	-
dos Santos et al. 2019 [67]	-	-	-	-	-	-	-	-	-	-	-	-
Moraes Losso et al. 2012 [68]	-	-	-	-	-	-	-	-	-	-	-	-
Leite et al. 2010 [69]	-	-	-	-	-	-	-	-	-	-	-	-
Proença et al. 2018 [70]	-	-	-	-	-	-	-	-	-	-	-	-
Rasuck et al. 2012 [71]	-	-	-	-	-	-	-	-	-	-	-	-
Santos et al. 2024 [72]	-	-	-	-	-	-	5	-	3	-	-	-
Afanador et al. 2022 [73]	-	-	-	-	-	-	-	-	-	-	-	-
Cardenas et al. 2008 [74]	-	-	-	-	-	-	-	-	-	-	-	-
Montenegro et al. 2006 [75]	-	-	-	-	-	-	-	-	-	-	-	-
Alvarez et al. 2021 [76]	-	-	-	-	-	-	-	-	-	-	-	-
Hurtado et al. 2015 [77]	-	-	-	-	-	-	-	-	-	-	-	-
MAríA Wielandt et al. 2017 [78]	4	11	-	-	-	-	-	-	-	-	-	-
Wielandt et al. 2020 [79]	-	-	-	-	-	-	-	-	-	-	-	-
Jordi et al. n.d. [80]	-	-	-	-	-	-	-	-	-	-	-	-
Ortiz et al. 2016 [81]	-	-	-	-	-	-	-	-	-	-	-	-
Perez-Mayoral et al. 2023 [82]	-	-	-	-	-	-	-	-	-	-	-	-
Sierra et al. 2021 [54]	-	-	-	-	-	-	-	-	-	-	-	-
Cruz-Correa et al. 2015 [55]	-	-	-	-	-	-	-	-	-	-	-	-
Vital et al. 2023 [83]	-	-	-	-	6	3	20	17	22	31	6	3
Antelo et al. 2019 [33]	-	-	-	-	-	-	-	-	-	-	-	-
Gupta et al. 2010 [27]	-	-	-	-	-	-	-	-	-	-	-	-

Altered markers were analyzed in 348 individuals from the MMRd group and 156 individuals assessed for MSI. In the MMRd group, the most frequent protein expression losses observed via IHC were MLH1/PMS2 in combination (156/348, 44.8%), followed by MSH2/MSH6 in combination (72/348, 20.7%). Other losses included MSH2 individually (40/348, 11.5%), MLH1 individually (35/348, 10.1%), PMS2 individually (23/348, 6.6%), and MSH6 individually (21/348, 6.0%). The least common marker was the combined loss of MSH2, MSH6, and MLH1 (1/348, 0.3%). Due to incomplete reporting of the microsatellites included in PCR-MSI panels or missing information in some studies, it was not possible to confirm the consistency of MSI results across studies.

### MMRd/MSI-H prevalence in Hispanic/Latinos

**Fig. 2:**
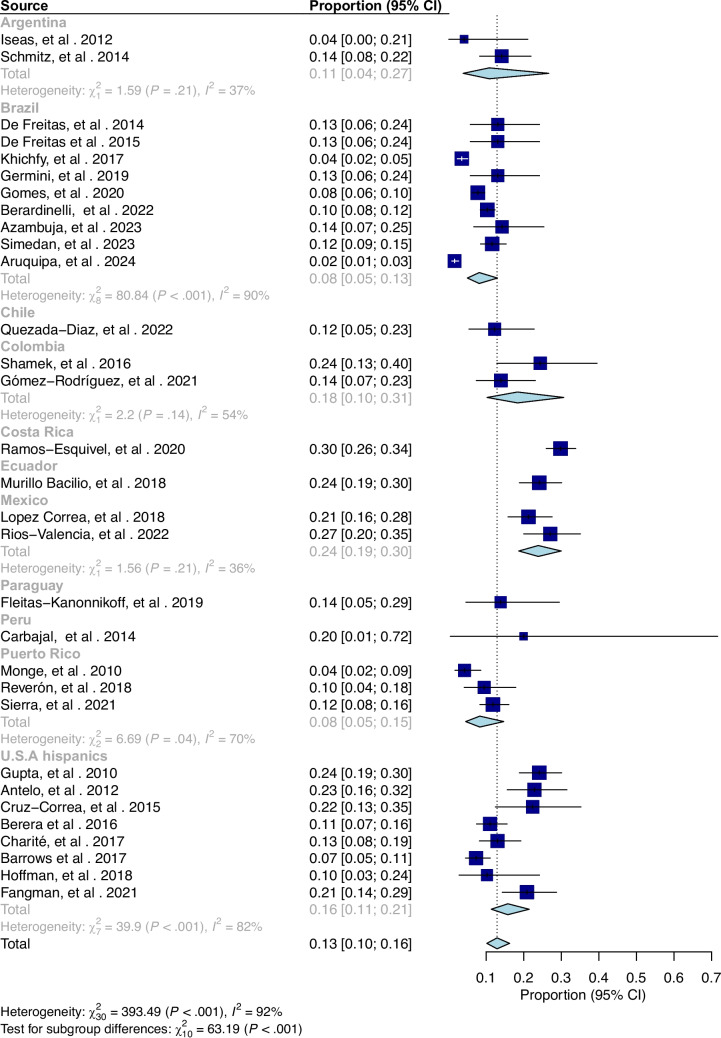
Meta-analysis of MMRd frequency

**Fig. 3:**
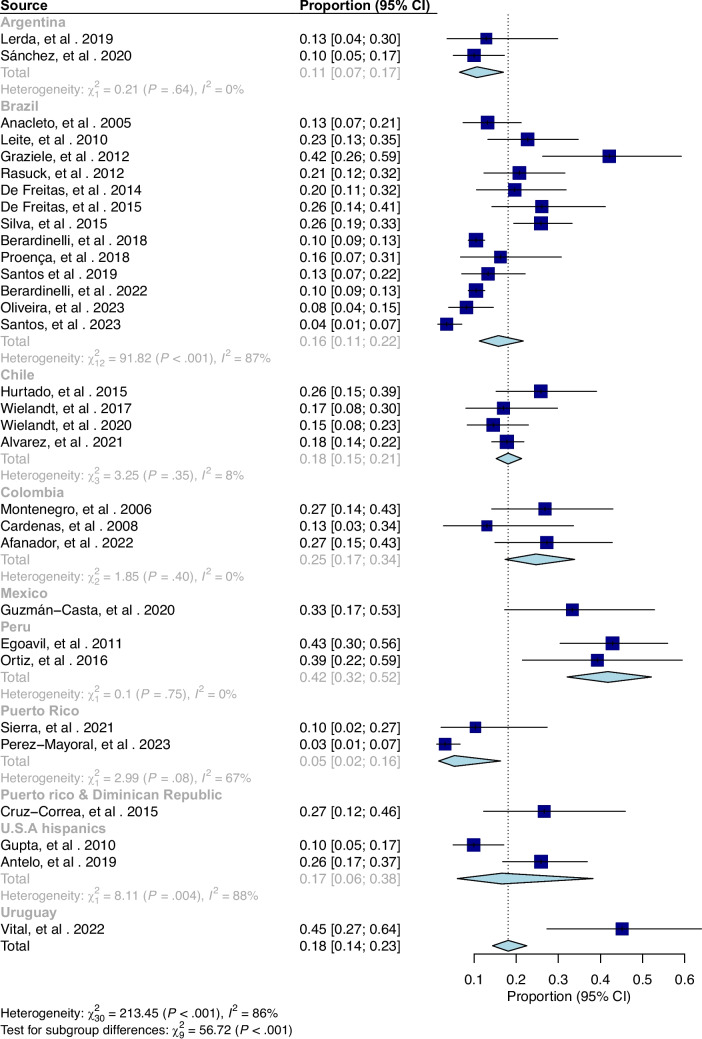
Meta-analysis of MSI-H frequency

Among the 28 articles reporting the prevalence of MMRd, a total of 6,475 individuals with available tests were included from Argentina, Brazil, Chile, Colombia, Costa Rica, Ecuador, Mexico, Paraguay, Peru, Puerto Rico, and Hispanic individuals in the U.S. The pooled MMRd prevalence was found to be 15% (95% CI: 10%−20%), with heterogeneity (I^2^ = 92%, P < 0.001) (Fig. 2). In the 28 articles that reported the prevalence of MSI-H, involving a total of 3,760 individuals with available tests from Argentina, Brazil, Chile, Colombia, Mexico, Peru, Puerto Rico, the Dominican Republic, Uruguay, and Hispanic individuals in the U.S, the pooled MSI-H prevalence was 18% (95% CI: 13%−24%) (Fig. 3).

**Fig. 4:**
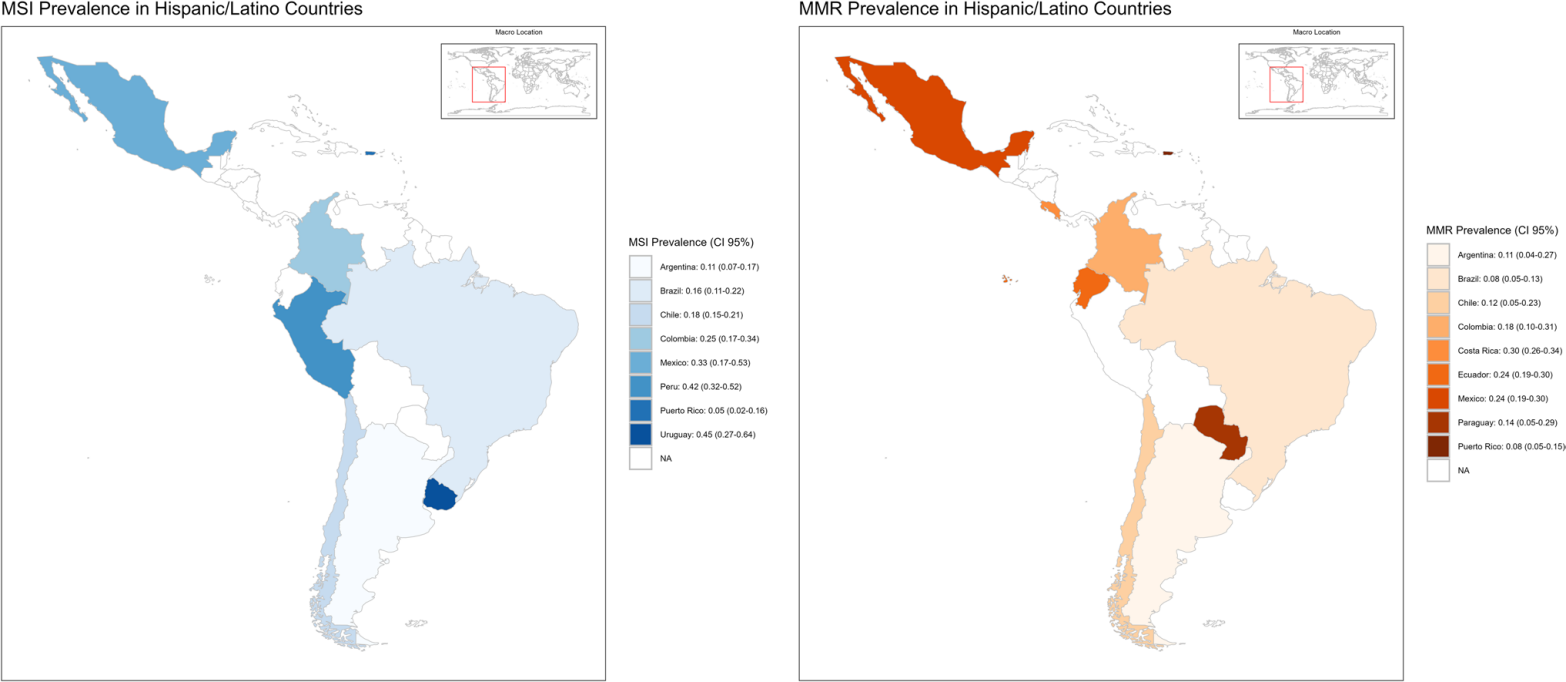
Prevalence of MMRd and MSI-H in Latin American countries

Country-based subgroup differences were statistically significant in both analyses (MMRd: χ^2^ = 63.19, P < 0.001; MSI-H: χ^2^ = 56.72, P < 0.001). Country-specific assessments revealed that Costa Rica had the highest MMRd prevalence at 30% (95% CI: 26%−34%), followed by Mexico at 24% (95% CI: 19%−30%). In contrast, Puerto Rico exhibited the lowest MMRd prevalence at only 8% (95% CI: 5%−15%). For MSI-H, Uruguay had the highest prevalence 45% (95% CI: 27%−64%), followed by Peru at 42% (95% CI: 32%−52%) and Mexico at 33% (95% CI: 17%−53%). Puerto Rico had the lowest MSI-H prevalence at 5% (95% CI: 2%−16%) (Fig. 4).

### Clinicopathological features correlated with MMRd/MSI-H status

A second meta-analysis was conducted to evaluate the relationship between clinicopathological features and MMRd/MSI-H (Supplementary Table 2). The results indicated that MSI-H was significantly associated with females, with an OR of 1.83 (1.40–2.39). Additionally, MMRd was significantly associated with tumors in the right colon compared to the rectum (OR: 1.73, 1.39–2.16) and with right-sided tumors compared to the left colon (OR: 5.65, 4.20–7.60). Similarly, MSI-H was more frequent in right-sided tumors compared to the left colon, with an OR of 8.16 (5.40–12.33).

In this study, no significant associations were observed between MMRd and sex or between MSI-H and tumor stage. This finding is likely due to the inclusion of these variables for descriptive purposes only, as they did not meet the inclusion criteria for the meta-analysis due to insufficient or inconsistent data across studies. Moreover, limitations in available data on histological type and TNM classification prevented a proper meta-analysis of these variables.

In the MMRd meta-analysis, the primary pooled proportion was 0.1315 (95% CI 0.1045–0.1642), with substantial heterogeneity (I^2^ = 89.6%; tau^2^ = 0.4289). Leave-one-out analysis showed only modest variation in the pooled estimate; the greatest change was observed after omission of Aruquipa et al. 2024 (Brazil) (33), which increased the pooled proportion to 0.1407 (95% CI 0.1151–0.1708) and reduced tau^2^ to 0.3624, while heterogeneity remained high.

In the MSI-H meta-analysis, the primary pooled proportion was 0.1946 (95% CI 0.1571–0.2385), also with substantial heterogeneity (I^2^ = 84.0%; tau^2^ = 0.3739). Sequential omission of individual studies again produced only modest changes in the pooled estimate. The largest change was observed after omission of Perez-Mayoral et al. 2023 (Puerto Rico) [82], which increased the pooled proportion to 0.2035 (95% CI 0.1671–0.2455) and reduced tau^2^ to 0.2974 and I^2^ to 81.0%. Influence diagnostics identified this study as the most influential observation in the MSI-H model; however, its exclusion did not materially change the overall interpretation, supporting the robustness of the pooled estimate despite persistent between-study heterogeneity.

### Quality assessment

A total of 52 articles underwent quality assessment. Most studies met the JBI criteria for cross-sectional (n = 41) and cohort studies (n = 11). However, among the cross-sectional studies, many did not account for confounding factors (n = 35), and a few did not report the statistical methods used (n = 4). In the cohort study group, some studies also failed to identify confounding factors (n = 5), and others did not address strategies for managing incomplete follow-up (n = 6). All included studies were classified as having a low risk of bias. Therefore, no studies were excluded based on quality assessment, and additional sensitivity analyses excluding high-risk studies were not considered necessary. The quality assessment for each study is detailed in Supplementary Table 3A and B.

## Discussion

Latin America is a highly diverse region with significant genetic variation, influenced by Native American (NAT), European (EUR), and African (AFR) ancestries. This makes it a unique area for studying colorectal cancer (CRC) biomarkers. Some studies suggest that genetic ancestry does not significantly affect the prevalence of MSI-H tumors [26, 84], while others report a higher prevalence of MSI-H tumors in European and African populations. A recent study by Matejcic et al. (2024), using whole-genome sequencing (NGS-MSI), found that NAT ancestry is associated with a lower frequency of MSI-H tumors compared to microsatellite-stable tumors (OR = 0.45, 95% CI = 0.21–0.99, p = 0.048) [32]. This highlights the relationship between genetic ancestry and tumor prevalence in the region.

In Hispanic/Latino populations, a recent meta-analysis of 201 studies from Europe, Asia, and North America reported prevalences of 11.7% for MMRd and 10.2% for MSI-H [85]. In our study of Hispanic/Latino populations, MMRd, detected by IHC, was present in 15% of cases, while MSI-H tumors were found in 18% of cases. These rates are higher than those reported by Ashktorab et al. (2016), who found an overall MSI prevalence of 12% across six studies focusing on Hispanic populations [26].

A country-by-country analysis showed notable differences in MMRd and MSI-H prevalence. This heterogeneity was also reflected in the meta-analysis (I^2^ = 86%), indicating substantial variability between studies. Costa Rica had the highest prevalence of MMRd at 30%, followed by Mexico at 24%, while Uruguay and Peru had the highest prevalence of MSI-H at (45% and 24% respectively). The high European ancestry in Costa Rica and Mexico, due to colonial and post-colonial migrations [42, 82, 84, 86], may explain their higher prevalence of MSI-H. On the other hand, both Mexico and Peru have high NAT ancestry [87–89], which was linked to lower MSI-H tumor frequencies in the study by Matejcic et al. [86]. These differences could be due to the methods used, as our study employed PCR-MSI and IHC-MSI, while Matejcic et al. used NGS-MSI. However, these country-specific differences highlight the genetic diversity within Hispanic/Latino populations and show the complexity of how ancestry influences MSI prevalence.

Several studies have suggested that differences in MMRd/MSI-H prevalence between Hispanic/Latino populations and other groups may be due to the higher rate of germline pathogenic/likely pathogenic (P/LP) variants in MMR genes (Lynch Syndrome) [27, 90, 91]. Hispanic/Latino individuals have a higher frequency of germline P/LP variants in MMR genes compared to Black/African American and White individuals (18.1% vs. 9.5% and 8.1%, respectively). Among individuals with CRC, Hispanic/Latino populations also show higher rates of P/LP variants compared to White, Asian, and Black individuals (18% vs. 16.2%, 12.6%, and 6.7%, respectively) [92]. A meta-analysis found that around 2.2% of all CRC cases carry germline P/LP variants in MMR genes across populations from Oceania, Europe, Asia, and North America [93]. In contrast, these variants account for 5–8% of CRC cases in Colombia [94] and 3.7% in Mexico [95]. In Brazil, germline P/LP variants in MMRd tumors were reported at 49% [42], much higher than the 33% observed in the U.S [96]. and 12.2% in Australia [97].

Additionally, Ricker et al. [91] reported that 13.0% of CRC tumors in Latino individuals exhibited MMRd, with 61.9% of these cases attributed to germline mutations in MMR genes. Across all age groups, CRC patients with germline variants, especially in MLH1, were often younger than 50 years, which is typical for Lynch syndrome. This suggests that sporadic MSI CRC may be less common in Hispanic individuals, and a significant proportion of MSI cases may be linked to Lynch syndrome [27].

Most studies reviewed here reported an average age of over 50 years for individuals with MMRd and MSI-H, except for Antelo M et al. [33] and Fangman BD et al. [59] in the MMR group (Table 1). Our study also found a significant association between female gender, right-sided tumors, and MSI-H, consistent with findings by Liang et al., who reported similar patterns in Asian populations (female: 54.1%, right-sided: 63.9% in IHC-MSI tumors) [98].

While the right-sided tumor pattern aligns with the clinical presentation of Lynch syndrome, the gender distribution contrasts with Lynch syndrome, which is more common in males, with higher lifetime risks (54–74%) compared to females (30–52%) [99]. The earlier onset of CRC in males compared to females further complicates the understanding of whether germline variants account for most MMRd/MSI-H cases in our cohort, making it difficult to generalize.

CIMP is the most frequent cause of sporadic MSI-H CRC [29]. Differences in MLH1 methylation between populations may help explain the variations in MMRd/MSI-H prevalence across ancestries and countries, supporting our findings. In Hispanic/Latino populations, MLH1 silencing remains high. Moreno-Ortiz et al. reported a methylation frequency of 25% in Mexican CRC patients, with a higher prevalence in women (71%) [100]. Similar frequencies have been reported in Brazil (23%), Colombia (24%), and Peru (38.4%) [43]. By contrast, a study in Slovakia found a higher MLH1 methylation prevalence of 45.8% [101], while studies in Asia showed much lower rates, such as 10.1% [102]. In the U.S., MLH1 promoter methylation was found in 20.3% of CRC patients, which is similar to Hispanic/Latino populations. Specifically, among U.S. Hispanic/Latino individuals, MLH1 silencing was reported at 12.6% [43].

Therefore, the higher prevalence of MMRd/MSI-H in the Hispanic/Latino populations in our study is likely due to both the higher frequency of Lynch syndrome and the significant prevalence of MLH1 promoter methylation in this group, including the U.S. Hispanic/Latino cohort. However, due to data limitations, we could not distinguish between sporadic and hereditary CRC cases.

The most common protein expression losses observed in this study via IHC-MSI were MLH1 and PMS2, followed by MSH2 and MSH6. This pattern aligns with findings from Asian populations [66, 86]. In 2019, Lizhu Chen et al. found that MLH1 and PMS2 were the most frequent protein loss combination in MMRd CRC cases, occurring in 41% of cases, while MSH2 and MSH6 losses were found in 20% of cases [103]. Less common combinations included losses of MSH6 and PMS2 or all four proteins [103].

Although MSI-H prevalence differs between Hispanic/Latino and Asian populations, our findings on protein expression losses are consistent. This may reflect shared genetic histories, as migration waves into East Asia, especially through Southeast Asia, contributed to the genetic diversity in both East Asian and Hispanic/Latino populations [43, 101, 102]. This genetic legacy may help explain the elevated rates of MLH1 silencing in Hispanic/Latino populations [104, 105] and the similarities in actionable somatic alterations between populations with NAT ancestry and South Asian patients [22].

No significant correlation was found between stage and MMRd/MSI-H due to data limitations, available studies indicate that 69.4% of MMRd/MSI-H CRC cases in Hispanic/Latino populations were diagnosed at stage I-II, while only 25.9% were stage IV. This is consistent with previous evidence showing that Hispanic/Latino individuals are more likely to be diagnosed with stage II and III CRC compared to Asian, White, and Black individuals [106]. Additionally, MSI-H tumors are more common in stage II and III (around 34%) and rare in stage IV (around 4%) [107]. This pattern may be explained by the less aggressive nature of MSI-H tumors, which have high mutational burdens that generate neoantigens, promoting immune cell infiltration and potentially limiting tumor growth and progression to later stages. However, Asian, White, and Black populations show a higher proportion of stage I tumors, suggesting that the higher prevalence of MSI-H in Hispanic/Latino populations may also be linked to later overall diagnosis [106].

The methodological quality of the included studies was assessed using the JBI critical appraisal tool for case series [30]. While most studies demonstrated acceptable methodological quality, several had notable limitations, including incomplete reporting of patient selection criteria, potential confounding factors, or a lack of standardized diagnostic approaches. These methodological differences may have contributed to the heterogeneity observed in prevalence estimates and should be considered when interpreting pooled results.

One key issue is the underrepresentation of many Hispanic/Latino countries, meaning the available data may not reflect the broader population, especially in regions with limited research or incomplete data collection. Additionally, many of the studies included did not evaluate populations using a complete set of MSI microsatellite markers or did not report this information. In several cases, MMRd/MSI-H status was assessed in only a subset of patients, limiting the inclusion of additional studies. These disparities highlight the need for standardized approaches to MMRd/MSI-H testing and reporting.

## Limitations

A potential limitation of this review is assay-related measurement bias, which may introduce some outcome misclassification across studies [108]. Importantly, this should be interpreted as a source of residual methodological variability rather than as a threat to the validity of the included estimates, because both MMR immunohistochemistry and PCR-based MSI testing are accepted, guideline-supported approaches for colorectal cancer and generally show high concordance [109]. However, these methods interrogate different levels of mismatch repair dysfunction, and discordant classifications may occur because of tumor heterogeneity, low tumor cell content, retained expression of non-functional proteins, unusual staining patterns, or other test-specific interpretative challenges [108, 110]. Therefore, a small proportion of the between-study variability may reflect differences in biomarker ascertainment rather than true underlying prevalence alone [110].

## Conclusion

This systematic review and meta-analysis provide a comprehensive evaluation of the prevalence, clinical features, and molecular markers associated with MMRd and MSI-H status among Hispanic/Latino populations. The overall prevalence of MMRd and MSI-H was 15% and 18%, respectively, with notable variations across countries. Costa Rica and Mexico demonstrated the highest MMRd prevalence, while Uruguay exhibited the highest MSI-H prevalence. Conversely, Puerto Rico consistently showed the lowest prevalence for both markers. These values differ from those seen in other populations and may be partly explained by differences in germline mutations, MLH1 methylation, and the tumor stage at diagnosis in Hispanic/Latino patients. Moreover, clinicopathological analyses revealed significant associations between MMRd/MSI-H and a predilection for tumors in the right colon. Additionally, MSI-H was more frequently observed in females. The participation and reporting of the rest of Latin American countries is warranted to address gaps in data and provide a deeper understanding of these molecular markers in diverse populations.

## Supplementary Information

Below is the link to the electronic supplementary material.

Supplementary file1 (DOCX 38 KB)

Supplementary file2 Leave-one-out sensitivity analysis and influence diagnostics for the MMRd prevalence meta-analysis (PDF 143 KB)

Supplementary file3 Leave-one-out sensitivity analysis and influence diagnostics for the MSI-H prevalence meta-analysis (PDF 163 KB)

Supplementary file4 (DOCX 15 KB)

Supplementary file5 (DOCX 15 KB)

Supplementary file6 (DOCX 34 KB)

## Data Availability

All relevant data are within the manuscript and its Supporting Information files.
