# Supplementary figures and images for: Microsatellite instability and mismatch repair deficiency prevalence among Hispanic/Latino individuals with colorectal cancer: a systematic review and meta-analysis
Source: Int J Colorectal Dis. 2026 May 21;41(1):118. doi: 10.1007/s00384-026-05146-2 (PMC13369342; doi:10.1007/s00384-026-05146-2)

## Leave-one-out sensitivity analysis

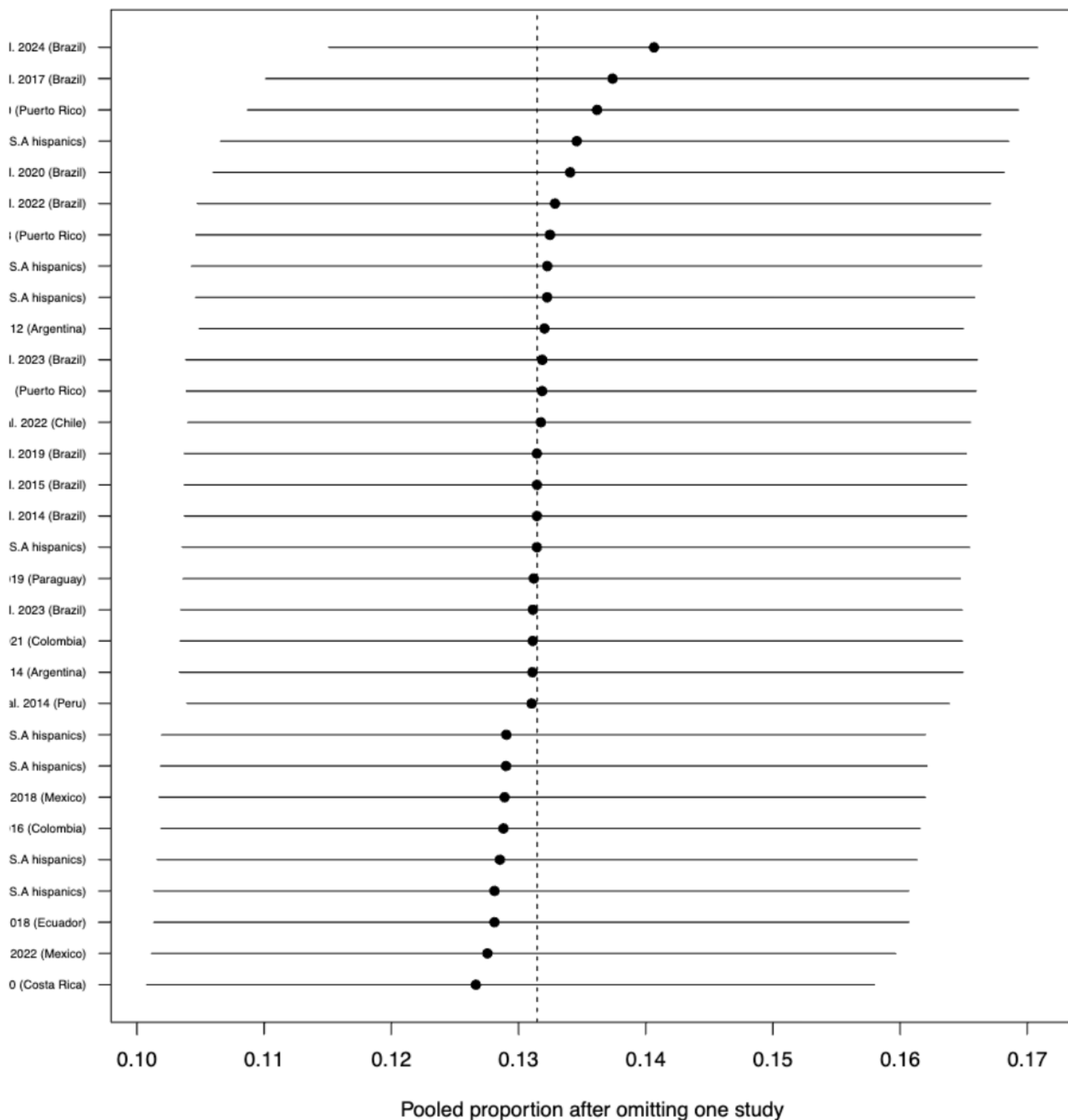

Supplement: Supplementary file 2 — Supplementary file2 Leave-one-out sensitivity analysis and influence diagnostics for the MMRd prevalence meta-analysis (PDF 143 KB) [file 384_2026_5146_MOESM2_ESM.pdf]

## Leave-one-out sensitivity analysis

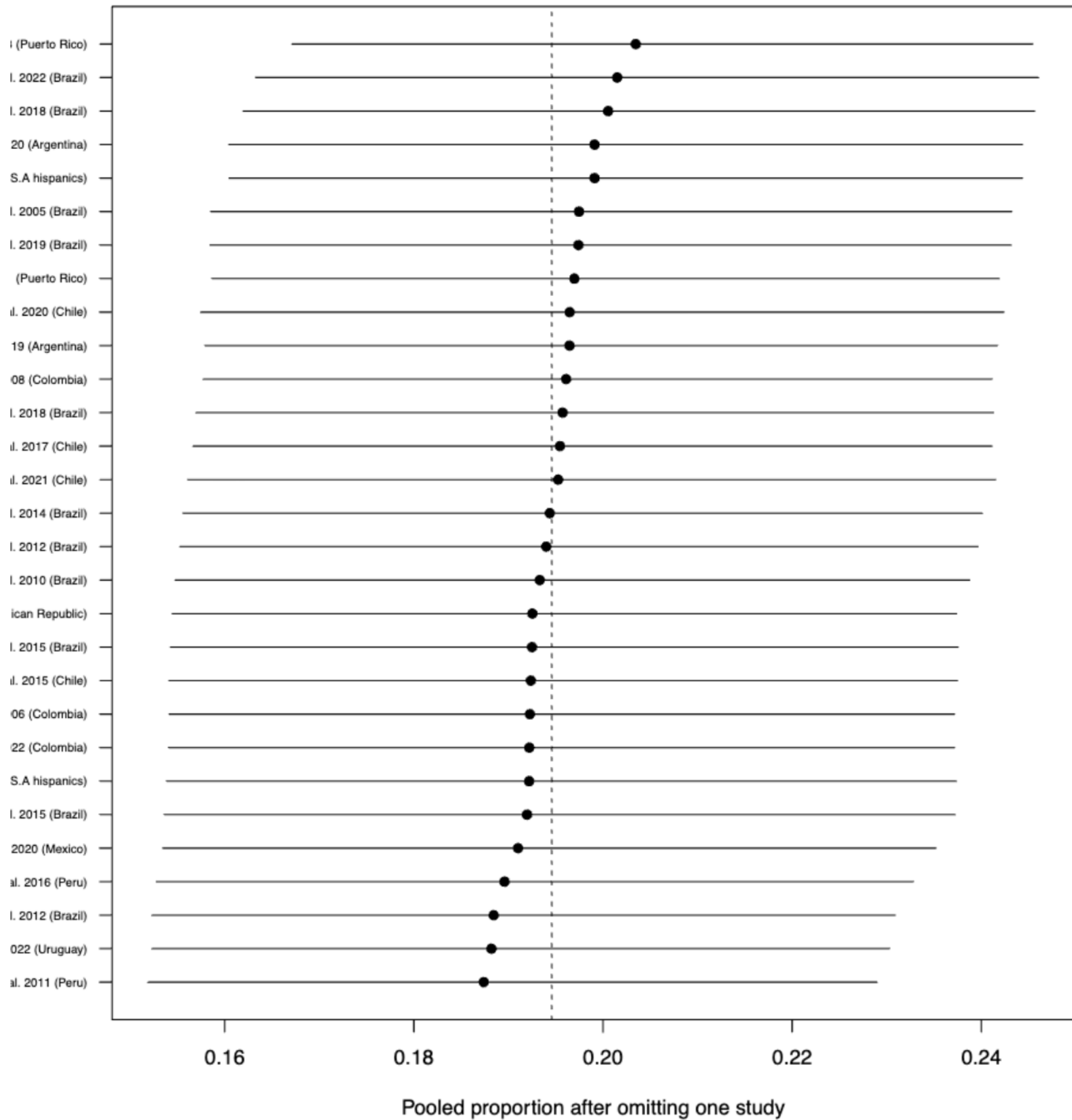

Supplement: Supplementary file 3 — Supplementary file3 Leave-one-out sensitivity analysis and influence diagnostics for the MSI-H prevalence meta-analysis (PDF 163 KB) [file 384_2026_5146_MOESM3_ESM.pdf]
