# Supplementary material for: Microsatellite instability and mismatch repair deficiency prevalence among Hispanic/Latino individuals with colorectal cancer: a systematic review and meta-analysis
Source: Int J Colorectal Dis. 2026 May 21;41(1):118. doi: 10.1007/s00384-026-05146-2 (PMC13369342; doi:10.1007/s00384-026-05146-2)
Supplement: Supplementary file 4 — Supplementary file4 (DOCX 15 KB) [file 384_2026_5146_MOESM4_ESM.docx]

| Database | Search strategy |
| --- | --- |
| PUBMED | ("caribbean region" OR "caribbean*" OR "latin american*" OR "latin-american*" OR "latin*" OR "hispanic*” OR “mexic*" OR "argentin*" OR "bolivia**" OR "brasil*" OR "brazil*" OR "chile*" OR "colombia*" OR "ecuador*" OR "guian*" OR "guyan*" OR "guiana francesca" OR "french guiana" OR "guyana francesa" OR "paragua*" OR "peru*" OR "surinam*" OR "urugua*" OR "venezuela*" OR "antiga e barbuda*" OR "antigua*" OR "barbuda" OR "lantigua y barbuda" OR "bahamas" OR "barbados" OR "beliz*" OR "costa ric*" OR "cuba*" OR "dominica*" OR "el salvador*" OR "granad*" OR "grenada" OR "guatemala*" OR "haiti*" OR "hondur*" OR "jamaica*" OR "nicaragua*" OR "panama*" OR "dominican republic*" OR "republica dominicana" OR "santalucia*" OR "saint lucia" OR "saocristovao e nevis" OR "san kittsnevis" OR "saint kitts" OR "nevis" OR "saovicente de grenadines" OR "san vicente y las grenadines" OR "saint vincent" OR "the grenadines" OR "trinidad et tobago" OR "trinidad y tabago" OR "trinidad" OR "tobago" OR "puerto ric*" OR "portorico" OR "guadeloup*" OR "guadalup*" OR "martiniqu*" OR "martini*" OR "saint martin*" OR "saint barthelem*" OR "america latina" OR "curaçao" OR "arub*" OR "anguilla") AND (("Mismatch repair" OR "Microsatellite Repeats" OR MMR OR dMMR OR "microsatellite instability" OR MSI))) AND (("Colon Neoplasm*" OR "colon cancer*" OR "colorectal Neoplasm*" OR "colorectal cancer*" OR "Colon Adenocarcinoma*" OR "Colorectal Adenocarcinoma*" OR "Cancer of the colon" OR "Colon tumor*" OR “colorectal tumor*") |
| EMBASE | ('colon neoplasm' OR 'colon cancer*' OR 'colorectal neoplasm*' OR 'colorectal cancer*' OR 'colon adenocarcinoma*' OR 'colorectal adenocarcinoma*' OR 'cancer of the colon' OR 'colon tumor*' OR 'colorectal tumor*') AND ('mismatch repair' OR 'mmr' OR 'dmmr' OR 'microsatellite instability' OR 'msi') AND ('caribbean region' OR 'caribbean*' OR 'latin american*' OR 'latin-american*' OR 'latin*' OR 'hispanic* mexic*' OR 'argentin*' OR 'bolivia**' OR 'brasil*' OR 'brazil*' OR 'chile*' OR 'colombia*' OR 'ecuador*' OR 'guian*' OR 'guyan*' OR 'guiana francesca' OR 'french guiana' OR 'guyana francesa' OR 'paragua*' OR 'peru*' OR 'surinam*' OR 'urugua*' OR 'venezuela*' OR 'antiga e barbuda*' OR 'antigua*' OR 'barbuda' OR 'lantigua y barbuda' OR 'bahamas' OR 'barbados' OR 'beliz*' OR 'costa ric*' OR 'cuba*' OR 'dominica*' OR 'el salvador*' OR 'granad*' OR 'grenada' OR 'guatemala*' OR 'haiti*' OR 'hondur*' OR 'jamaica*' OR 'nicaragua*' OR 'panama*' OR 'dominican republic*' OR 'republica dominicana' OR 'santalucia*' OR 'saint lucia' OR 'saocristovao e nevis' OR 'san kittsnevis' OR 'saint kitts' OR 'nevis' OR 'saovicente de grenadines' OR 'san vicente y las grenadines' OR 'saint vincent' OR 'the grenadines' OR 'trinidad et tobago' OR 'trinidad y tabago' OR 'trinidad' OR 'tobago' OR 'puerto ric*' OR 'portorico' OR 'guadeloup*' OR 'guadalup*' OR 'martiniqu*' OR 'martini*' OR 'saint martin*' OR 'saint barthelem*' OR 'america latina' OR 'curaçao' OR 'arub*' OR 'anguilla') |
| LILACS | ( ("mismatch repair" OR mmr OR dmmr OR "microsatellite instability" OR msi) AND ("colon neoplasm*" OR "colon cancer*" OR "colorectal neoplasm*" OR "colorectal cancer*" OR "colon adenocarcinoma*" OR "colorectal adenocarcinoma*" OR "cancer of the colon" OR "colon tumor*" OR "colorectal tumor*") AND (hispanic* OR latin* OR mexico OR argentina OR bolivia OR brasil OR brazil OR chile OR colombia OR ecuador OR guiana OR guyana OR "French guiana" OR paraguay OR peru OR suriname OR uruguay OR venezuela OR antigua OR barbuda OR bahamas OR barbados OR belize OR costa rica OR cuba OR dominica OR "el salvador" OR granada OR grenada OR guatemala OR haiti OR honduras OR jamaica OR nicaragua OR panama OR "dominican republic" OR "republica dominicana" OR santalucia OR santalucia OR saint lucia OR saint kitts OR nevis OR "saint vincent" OR the grenadines OR trinidad OR tobago OR "Puerto rico" OR portorico OR guadeloupe OR guadalupe OR martinique OR martini OR "saint martin")) |
| SCOPUS | ("caribbean region" OR "caribbean*" OR "latin american*" OR "latin-american*" OR "latin*" OR "hispanic*" OR "mexic*" OR "argentin*" OR "bolivia*" OR "brasil*" OR "brazil*" OR "chile*" OR "colombia*" OR "ecuador*" OR "guian*" OR "guyan*" OR "guiana francesca" OR "french guiana" OR "guyana francesa" OR "paragua*" OR "peru*" OR "surinam*" OR "urugua*" OR "venezuela*" OR "antiga e barbuda*" OR "antigua*" OR "barbuda" OR "lantigua y barbuda" OR "bahamas" OR "barbados" OR "beliz*" OR "costa ric*" OR "cuba*" OR "dominica*" OR "el salvador*" OR "granad*" OR "grenada" OR "guatemala*" OR "haiti*" OR "hondur*" OR "jamaica*" OR "nicaragua*" OR "panama*" OR "dominican republic*" OR "republica dominicana" OR "santalucia*" OR "saint lucia" OR "saocristovao e nevis" OR "san kittsnevis" OR "saint kitts" OR "nevis" OR "saovicente de grenadines" OR "san vicente y las grenadines" OR "saint vincent" OR "the grenadines" OR "trinidad et tobago" OR "trinidad y tabago" OR "trinidad" OR "tobago" OR "puerto ric*" OR "portorico" OR "guadeloup*" OR "guadalup*" OR "martiniqu*" OR "martini*" OR "saint martin*" OR "saint barthelem*" OR "america latina" OR "curaçao" OR "arub*" OR "anguilla") AND (("Mismatch repair" OR "Microsatellite Repeats" OR MMR OR dMMR OR "microsatellite instability" OR MSI)) AND (("Colon Neoplasm*" OR "colon cancer*" OR "colorectal Neoplasm*" OR "colorectal cancer*" OR "Colon Adenocarcinoma*" OR "Colorectal Adenocarcinoma*" OR "Cancer of the colon" OR "Colon tumor*" OR "colorectal tumor*")) |
| Web of science | ("caribbean region" OR "caribbean*" OR "latin american*" OR "latin-american*" OR "latin*" OR "hispanic*" OR "mexic*" OR "argentin*" OR "bolivia*" OR "brasil*" OR "brazil*" OR "chile*" OR "colombia*" OR "ecuador*" OR "guian*" OR "guyan*" OR "guiana francesca" OR "french guiana" OR "guyana francesa" OR "paragua*" OR "peru*" OR "surinam*" OR "urugua*" OR "venezuela*" OR "antiga e barbuda*" OR "antigua*" OR "barbuda" OR "lantigua y barbuda" OR "bahamas" OR "barbados" OR "beliz*" OR "costa ric*" OR "cuba*" OR "dominica*" OR "el salvador*" OR "granad*" OR "grenada" OR "guatemala*" OR "haiti*" OR "hondur*" OR "jamaica*" OR "nicaragua*" OR "panama*" OR "dominican republic*" OR "republica dominicana" OR "santalucia*" OR "saint lucia" OR "saocristovao e nevis" OR "san kittsnevis" OR "saint kitts" OR "nevis" OR "saovicente de grenadines" OR "san vicente y las grenadines" OR "saint vincent" OR "the grenadines" OR "trinidad et tobago" OR "trinidad y tabago" OR "trinidad" OR "tobago" OR "puerto ric*" OR "portorico" OR "guadeloup*" OR "guadalup*" OR "martiniqu*" OR "martini*" OR "saint martin*" OR "saint barthelem*" OR "america latina" OR "curaçao" OR "arub*" OR "anguilla") AND ("Mismatch repair" OR "Microsatellite Repeats" OR "MMR" OR "dMMR" OR "microsatellite instability" OR "MSI") AND ("Colon Neoplasm*" OR "colon cancer*" OR "colorectal Neoplasm*" OR "colorectal cancer*" OR "Colon Adenocarcinoma*" OR "Colorectal Adenocarcinoma*" OR "Cancer of the colon" OR "Colon tumor*" OR "colorectal tumor*") |

Supplementary table 1. Search strategy
