# Supplementary material for: Microsatellite instability and mismatch repair deficiency prevalence among Hispanic/Latino individuals with colorectal cancer: a systematic review and meta-analysis
Source: Int J Colorectal Dis. 2026 May 21;41(1):118. doi: 10.1007/s00384-026-05146-2 (PMC13369342; doi:10.1007/s00384-026-05146-2)
Supplement: Supplementary file 5 — Supplementary file5 (DOCX 15 KB) [file 384_2026_5146_MOESM5_ESM.docx]

**Supplementary Table 2. Association of MMRd/MSI-H status with clinicopathological features**

| **Comparison** | **Number of studies** | **Odds Ratio** | **95% IC** | **p** |
| --- | --- | --- | --- | --- |
| MSI-H status and sex | 8 | 2.47 | (1.73-3.53) | <0.00001 |
| MMRd status and sex | 10 | 0.90 | 0.72-1.13 | 0.38 |
| MMRd and tumor location (colon vs. rectum) | 11 | 1.73 | 1.39-2.16 | <0.00001 |
| MMRd in right-sided vs. left-sided tumors | 6 | 5.65 | 4.20-7.60 | <0.00001 |
| MSI-H in right-sided vs. left-sided tumors | 4 | 9.53 | 4.47-20.32 | <0.00001 |
| MSI-H status and tumor stage (I-II vs. III-IV) | 6 | 1.08 | 0.65-1.77 | 0.77 |
