# Supplementary material for: Microsatellite instability and mismatch repair deficiency prevalence among Hispanic/Latino individuals with colorectal cancer: a systematic review and meta-analysis
Source: Int J Colorectal Dis. 2026 May 21;41(1):118. doi: 10.1007/s00384-026-05146-2 (PMC13369342; doi:10.1007/s00384-026-05146-2)
Supplement: Supplementary file 6 — Supplementary file6 (DOCX 34 KB) [file 384_2026_5146_MOESM6_ESM.docx]

| Date of assesment | Study | Were the criteria for inclusion in the sample clearly defined? | Were the study subjects and the setting described in detail? | Was the exposure measured in a valid and reliable way? | Were objective, standard criteria used for measurement of the condition? | Were confounding factors identified? | Were strategies to deal with confounding factors stated? | Were the outcomes measured in a valid and reliable way? | Was appropriate statistical analysis used? | Overall |
| --- | --- | --- | --- | --- | --- | --- | --- | --- | --- | --- |
| 04/09/2024 | Charité et al. 2017 | Y | Y | Y | Y | Y | Y | Y | Y | INCLUDED |
| 04/09/2024 | Hoffman, SJ 2018 | Y | Y | Y | Y | N | N | Y | Y | INCLUDED |
| 04/09/2024 | de Freitas et al. 2015 | Y | Y | Y | Y | Y | N | Y | Y | INCLUDED |
| 04/09/2024 | Proença MA 2018 | Y | Y | Y | Y | N | N | Y | Y | INCLUDED |
| 04/09/2024 | Lerda et al. 2019 | Y | Y | Y | Y | N | N | Y | NA | INCLUDED |
| 04/09/2024 | Gomes et al. 2020 | Y | Y | Y | Y | N | N | Y | Y | INCLUDED |
| 04/09/2024 | Simedan et al 2023 | Y | Y | Y | Y | Y | Y | Y | Y | INCLUDED |
| 04/09/2024 | Schmitz et al. 2014 | Y | Y | Y | Y | NA | NA | Y | NA | INCLUDED |
| 04/09/2024 | Lopez Correa et al 2018 | Y | Y | Y | Y | NA | NA | Y | NA | INCLUDED |
| 04/09/2024 | Reverón et al 2018 | Y | Y | Y | Y | N | N | Y | Y | INCLUDED |
| 04/09/2024 | Vital et al 2022 | Y | Y | Y | Y | N | N | Y | Y | INCLUDED |
| 04/09/2024 | Egoavil et al 2011 | Y | Y | Y | Y | N | N | Y | Y | INCLUDED |
| 04/09/2024 | Shamek et al. 2016 | Y | Y | Y | Y | N | N | Y | Y | INCLUDED |
| 04/09/2024 | Sierra et al. 2021 | Y | Y | Y | Y | Y | Y | Y | Y | INCLUDED |
| 04/09/2024 | Gómez-Rodríguez et al. 2021 | Y | Y | Y | Y | N | N | Y | Y | INCLUDED |
| 04/09/2024 | Rios-Valencia et al. 2022 | Y | Y | Y | Y | N | N | Y | Y | INCLUDED |
| 04/09/2024 | dos Santos et al. 2019 | Y | Y | Y | Y | Y | Y | Y | Y | INCLUDED |
| 05/09/2024 | Graziele, et al. 2012 | Y | Y | Y | Y | N | N | Y | Y | INCLUDED |
| 05/09/2024 | Ortiz, et al. 2016 | Y | Y | Y | Y | N | N | Y | Y | INCLUDED |
| 05/09/2024 | Perez-Mayoral, et al. 2023 | Y | Y | Y | Y | N | N | Y | Y | INCLUDED |
| 05/09/2024 | Silva, et al. 2015 | Y | Y | Y | Y | N | N | Y | Y | INCLUDED |
| 05/09/2024 | Antelo M, et al. 2019 | Y | Y | Y | Y | N | N | Y | Y | INCLUDED |
| 05/09/2024 | Berardinelli GN, et al. 2018 | Y | Y | Y | Y | N | N | Y | Y | INCLUDED |
| 05/09/2024 | Cardenas, Wilmer et al. 2008 | Y | Y | Y | Y | N | N | Y | Y | INCLUDED |
| 05/09/2024 | Montenegro M, Yenny et al. 2006 | Y | Y | Y | Y | N | N | Y | Y | INCLUDED |
| 05/09/2024 | Leite SM, et al. 2010 | Y | Y | Y | Y | N | N | Y | Y | INCLUDED |
| 05/09/2024 | Rasuck CG, et al. 2012 | Y | Y | Y | Y | N | N | Y | Y | INCLUDED |
| 05/09/2024 | Sunagua Aruquipa M, et al. 2024 | Y | Y | Y | Y | N | N | Y | Y | INCLUDED |
| 05/09/2024 | Afanador CH, et al. 2022 | Y | Y | Y | Y | N | N | Y | Y | INCLUDED |
| 05/09/2024 | Sánchez AG, et al. 2020 | Y | Y | Y | Y | N | N | Y | Y | INCLUDED |
| 05/09/2024 | Cruz-Correa M, et al. 2015 | Y | Y | Y | Y | N | N | Y | Y | INCLUDED |
| 05/09/2024 | Anacleto C, et al. 2005 | Y | Y | Y | Y | Y | Y | Y | Y | INCLUDED |
| 05/09/2024 | Oliveira JW, et al. 2023 | Y | Y | Y | Y | N | N | Y | Y | INCLUDED |
| 05/09/2024 | Guzmán-Casta, et al. 2020 | Y | Y | Y | Y | N | N | Y | Y | INCLUDED |
| 05/09/2024 | Wielandt, et al . 2017 | Y | Y | Y | Y | N | N | Y | Y | INCLUDED |
| 05/09/2024 | Wielandt, et al. 2020 | Y | Y | Y | Y | N | N | Y | Y | INCLUDED |
| 11/09/2024 | Barrows et al. 2017 | Y | Y | Y | Y | N | N | Y | Y | INCLUDED |
| 11/09/2024 | Berera et al. 2016 | Y | Y | Y | Y | Y | Y | Y | Y | INCLUDED |
| 23/09/2024 | Ñique Carbajal, et al. 2014 | Y | Y | Y | Y | N | N | Y | Y | INCLUDED |
| 02/12/2024 | Murillo Bacilio MdR, et al 2018 | Y | Y | Y | Y | N | N | Y | Y | INCLUDED |

**Supplementary Table 3A**. Quality assessment of cross-sectional studies included

| Date | Study | 1. Were the two groups similar and recruited from the same population? | 2. Were the exposures measured similarly to assign people to both exposed and unexposed groups? | 3. Was the exposure measured in a valid and reliable way? | 4. Were confounding factors identified? | 5. Were strategies to deal with confounding factors stated? | 6. Were the groups/participants free of the outcome at the start of the study (or at the moment of exposure)? | 7. Were the outcomes measured in a valid and reliable way? | 8. Was the follow up time reported and sufficient to be long enough for outcomes to occur? | 9. Was follow up complete, and if not, were the reasons to loss to follow up described and explored? | 10. Were strategies to address incomplete follow up utilized? | 11. Was appropriate statistical analysis used? | Overall appraisal: |
| --- | --- | --- | --- | --- | --- | --- | --- | --- | --- | --- | --- | --- | --- |
| 04/09/2024 | Santos, FA, et al 2023 | Y | Y | Y | Y | Y | Y | Y | Y | Y | Y | Y | INCLUDED |
| 04/09/2024 | Quezada-Diaz, F 2022 | Y | Y | Y | N | N | Y | Y | Y | Y | Y | Y | INCLUDED |
| 04/09/2024 | Alex, Alexandra Khichfy 2017 | Y | Y | Y | Y | N | Y | Y | Y | N | N | Y | INCLUDED |
| 04/09/2024 | Fangman et al. 2021 | Y | Y | Y | Y | Y | Y | Y | U | U | N | Y | INCLUDED |
| 04/09/2024 | Azambuja et al 2023 | Y | Y | Y | Y | Y | Y | Y | Y | N | Y | Y | INCLUDED |
| 04/09/2024 | Ramos-Esquivel et al. 2020 | Y | Y | Y | Y | Y | Y | Y | Y | Y | Y | Y | INCLUDED |
| 04/09/2024 | Jesus-Monge et al. 2010 | Y | Y | Y | N | N | Y | Y | Y | Y | Y | Y | INCLUDED |
| 05/09/2024 | Gupta, et al. 2010 | Y | Y | Y | N | N | Y | Y | Y | Y | Y | Y | INCLUDED |
| 05/09/2024 | Fleitas-Kanonnikoff, et al. 2019 | Y | Y | Y | N | N | Y | Y | Y | Y | Y | Y | INCLUDED |
| 05/09/2024 | Hurtado, Claudia et al. 2015 | Y | Y | Y | N | N | Y | Y | Y | Y | N | Y | INCLUDED |
| 05/09/2024 | Germini, et al. 2019 | Y | Y | Y | N | N | Y | Y | Y | Y | N | Y | INCLUDED |
| 05/09/2024 | Alvarez K, et al. 2021 | Y | Y | Y | Y | Y | Y | Y | U | U | N | Y | INCLUDED |

**Supplementary Table 3B**. Quality assessment of cohort studies included
